# Housing and psychosocial factors associated with mental health in children aged 6–12 years from homeless families in the Greater Paris area, France: the ENFAMS cross-sectional study

**DOI:** 10.3389/frcha.2023.1136597

**Published:** 2023-06-20

**Authors:** Mégane Estevez, Nicolas Oppenchaim, Alexandra Descarpentrie, Lison Rambliere, Caroline Douay, Cédric Galera, Stéphanie Vandentorren

**Affiliations:** ^1^ Inserm UMR 1219, Bordeaux Population Health Research Center, PHARes Team, University of Bordeaux, Bordeaux, France; ^2^ UMR CITERES 7324, University of Tours, Tours, France; ^3^ Université Paris Cité and Université Sorbonne Paris Nord, Inserm, INRAE, Center for Research in Epidemiology and StatisticS (CRESS), Paris, France; ^4^ Observatoire du Samusocial de Paris, Paris, France; ^5^ Department of Child and Adolescent Psychiatry, Centre Hospitalier Charles Perrens, Bordeaux, France; ^6^ Direction Scientifique et International, Santé publique France, Saint Maurice, France; ^7^ Department of Health, Institut Convergences Migrations/CNRS, Aubervilliers, France

**Keywords:** homeless families, psychosocial factors, children’s mental health, dominic interactive, social inequalities in health, housing, structural social determinants, social exclusion

## Abstract

**Objective:**

This study aimed to identify the housing and psychosocial factors associated with mental health disorders in children aged 6–12 years living in sheltered homeless families in the Greater Paris area (France), with a view to guiding the development of actions that could improve their mental health.

**Method:**

The cross-sectional study ENFAMS (“Enfants et familles sans logement”) was conducted between January and May 2013 on a random sample of sheltered homeless families in the Greater Paris area using face-to-face questionnaires administered by trained interviewers and psychologists in 17 languages. The questionnaires collected data on socio-demographics, living conditions, and health characteristics for the child and one of the parent selected. Mental health disorders were assessed in 198 children using the Dominic Interactive tool. Statistical analyses were performed using multiple linear regression on complete data.

**Results:**

The Dominic Interactive mean score was 28.8 (SD = 17.5), and it showed that 25.3% of the children had a possible or probable mental health disorder. Factors associated with higher children total difficulties scores, as measured by the Dominic Interactive, were parents' irregular administrative status, the child sleeping in the parents' bed, the child having been hospitalized in the past 12 months, and the child being bullied at school.

**Conclusion:**

Our findings highlight the importance of psychosocial determinants in children's mental health, and underline the need for prevention actions for homeless families which focus on improving living, schooling, and healthcare conditions, especially greater access to mental health care.

## 1. Introduction

Housing is a structural health determinant and human right that intersects with or influences different individuals' life dimensions. Housing can profoundly affect both physical and mental health at all levels through direct (structural features of the home) and indirect pathways (affordability, stability, frequent moves, homelessness). Poor housing conditions have been associated with respiratory diseases due to poor indoor air quality and mold, cognitive delays in children from exposure to lead accidents and injuries because of structural deficiencies (1). Poor housing quality and instability have also been associated with mental health issues as it disrupts work, school, day care arrangements, as well as social networks of both parents and children (2).

Homelessness is a complex societal issue that occur at micro, meso, and macro-levels of society. At the macro level, structural economic and social factors such as lack of available low-cost housing, poor economic conditions, discrimination and unequal access to distribution of financial, educational, and employment as well as health resources led to homelessness (3–6). The meso-level factors include lack of community support and rental supplements and adverse events such as family breakdown although at the micro-level, individual's factors include uncertain physical and mental health, addictions or history of migration (7). All these factors drive individuals and families (including their children) into homelessness (3). People can be categorized as homeless if they are living on the streets, moving between temporary shelters, including houses of friends, family, and emergency accommodation or have no permanent house or place to live safely (8). The number of homeless families has substantially increased throughout Europe in recent years. This phenomenon has become a major social and public health issue, and families represent a significant and unrecognized portion of the homeless population since 2010. However, there is a lack of visibility about this issue, and related literature is scarce (9, 10).

In France, approximately 30,000 children were living in a homeless family at the beginning of 2012, according to the French National Institute for Statistics and Economic Studies (*Institut national de la statistique et des études économiques*, INSEE). INSEE considers a person homeless if, on the night before the yearly INSEE survey, the person used a shelter service or slept in a place not intended for habitation (e.g., street, makeshift shelter) (11, 12). In November 2019, several associations warned that 500 to 700 children were denied access to emergency accommodation every night in the city of Paris, while 20,000 children were housed in welfare hotels in very precarious conditions in the Greater Paris area (13).

The role of housing in child development and health outcomes is crucial. Homelessness, poverty and poor living conditions make children particularly vulnerable to health problems including mental health (14). Specifically poor housing conditions threaten their safety, and impact their short-term and long-term development, as living in poor and/or overcrowded housing has a life-long impact on their health and well-being (15). Previous studies have found that overcrowded conditions were associated to slow growth in childhood, and an increased risk of coronary heart disease in later life (16). Overcrowding is linked to delayed cognitive development, and homelessness to delayed development in communication skills (17). Children living in poor or overcrowded conditions are more likely to suffer from infectious diseases (15) (meningitis (18), tuberculosis (19)), or respiratory disease (coughing and asthmatic wheezing) and three to four time more likely to have mental health problems (anxiety and depression). Homeless children are two to three times more likely to miss school due to the disruption caused moving between temporary accommodations or due to illnesses (15). To date, the only survey in France focusing on homeless families is ENFAMS, conducted by the “Observatoire du Samu Social de Paris” in 2013. Analyses highlighted various interacting difficulties linked to these families' vulnerability, notably elements linked to homelessness (residential instability, housing conditions, etc.) and to their migration status (lack of knowledge of the French health care system, language barriers, etc.). Overall, the survey revealed that homeless families had poor housing conditions, accommodation instability, a lack of resources, health problems, and limited access to healthcare services (14).

Assessing mental health by the total difficulties scores from the Strength and Difficulties Questionnaire (SDQ) reported by mothers, Roze et al. found that on average, homeless children in France were more likely to have mental health problems than children in the general population (20). They also identified social, environmental, individual and family factors associated with emotional and behavioral difficulties in children. Specifically, children's difficulties were associated with residential mobility, their health, having overweight, their sleep patterns, disliking their shelter, being bullied in school, their parents' region of birth, and suicidal risk in mothers.

As mentioned above, Roze et al. used the mother-reported SDQ for their analysis; it is well known that informants (e.g., parents, children) may differ in terms of their perceptions and awareness of mental health problems of others depending on the context, and probably depending on their own mental health. In general, children are more likely to report internalized disorders (i.e., anxiety, depressive symptoms) whereas their parents are more likely to report externalized disorders (impulsivity, agitation) (21). This may be related to the fact that internalized disorders are most often characterized by quiet, internal distress sometimes referred to as “intropunitive,” rather than externalized disorders that referred to overtly, socially negative, or disruptive behavior (22).

Mental health is important to overall health. When mental health problems occur during critical periods of development, such as early childhood, they are particularly damaging. These problems are described as severe changes in the ways children learn, behave, and manage their emotions, resulting in distress and difficulty coping with life stressors (23). If they did not receive appropriate support and care, mental health problems could become chronic and could interact with different children's life dimensions (23). Without early diagnosis and treatment, children with mental health problems may have problems at home, at school, and in forming friendships (23) that can persist over a longer time. Homelessness is linked to delayed development in communication skills (17). Homeless children are more likely to have behavioral problems such as aggression, hyperactivity and impulsivity factors that compromise academic achievement and relationships with peers and teachers (15).

To date, no study in France has evaluated homeless children's mental health using a tool which collects responses directly from children themselves. Such an evaluation is necessary not only to balance parents’ perceptions of their child's mental health, but also to better take into account children's voice and to give them the opportunity to assess directly their own mental health. Furthermore, studies found that child self-reports may be better predictors of later mental health diagnosis compared to parental reports (24). Finally, its simple and rapid use facilitates the clinical evaluation of children by professionals, allows the evaluation of intervention programs and promotes early intervention (25). However, few studies using child self-reports have targeted specific or vulnerable populations such as homeless children and focus on structural social determinants of their mental health. We hypothesize that structural social determinants play a crucial role in child's mental health.

The main objective of the present study was to identify structural social determinants of mental health such as housing and sheltering conditions, demographics (family structure, administrative status) and other psychosocial factors associated with child reported poor mental health in children aged 6–12 years living in homeless families in the Greater Paris area.

## 2. Materials and methods

### 2.1. Study design and population

This present study was based on data from the cross-sectional ENFAMS survey (*Enfants et familles sans logement*) which was conducted between January and May 2013 by l'Observatoire du Samusocial de Paris. Following guidelines from INSEE, a person is considered to be homeless on any given day if he or she spent the previous night in a sheltered accommodation or slept in a place not intended for living (on the street, in a squat, vehicles, abandoned buildings, public places including streets, parks etc.) (26).

The survey was designed to assess the health status of homeless mothers and children in Greater Paris area (approximately 12 million inhabitants) according to various structural social determinants. A special focus was placed on living and housing conditions (i.e., residential instability, food insecurity) in regard to mental health of mothers and children.

For ENFAMS, an eligible family was defined as comprising at least one parent (>18 years old) with at least one child younger than 13 years, who (the parent) spoke one of the 17 languages of the survey and was able to provide written consent to participate. The survey used a time-location sampling design. In the first stage, all housing facilities (emergency shelters, long-term re-habilitation centers, social hostels, and centers for asylum seekers) for homeless families in the Paris area were listed. A total of 251 housing facilities in the Paris area were selected for the ENFAMS survey, with an 82% participation rate. At the second stage, in these housing facilities, 1,238 families were selected to participate (with a 65% participation rate), resulting in a sample of 801 families. At the third stage, for each family, one child among all those younger than 13 years and the mother (or father, if the mother was absent) were randomly selected to participate in the survey.

The present study included all school-age children (i.e., 6–12 years), whose parents were born abroad, and who were evaluated by the Dominic Interactive (DI) as part of ENFAMS. Children of French parents were excluded as the small number (*N* = 5) involved would have made comparison with children of migrants difficult. A total of 198 children were included in the descriptive analyses and 128 children were included in the multivariate analysis; further details on the rationale for the selection of the study participants can be found in the Results section.

### 2.2. Data collection

Data for the ENFAMS survey were collected by a pair of interviewers who administered two paper-based face-to-face questionnaires (available in 17 languages) to the parent (preferably the mother). One questionnaire focused on the family as a whole (family characteristics, living conditions, health and health care use of the adult interviewed); the second questionnaire concerned one of the children in the family (the one selected at random), and included questions on the child's social relationships and health. The child, aged 6 to 12, was also interviewed directly by the psychologist-interviewer, at the same time as the parent was interviewed. This paper-based questionnaire focused on the child's relationship to housing and school, as well as on his or her social relationships and leisure activities. Then, the 6–12-year-old child, was assessed by the DI which was not in paper form but rather digital as it is a graphical material in a format similar to a computer game.

#### 2.2.1. Children's mental health

The DI is an international self-administered computerized questionnaire of children's mental health. It is employed for the early detection of mental health disorders in children between the ages of 6 and 12 years, by focusing on internalized and externalized problems. It diagnoses the following seven mental health disorders: specific phobias, separation anxiety disorder, generalized anxiety disorder, major depression/dysthymia (internalized problems), oppositional defiant disorder, conduct disorder, and attention deficit hyperactivity disorder (externalized problems). The tool assesses 62 of the 66 symptoms related to these seven mental health disorders as listed in the Diagnostic and Statistical Manual—Revision 4 (DSM-IV) (27). The tool's algorithm calculates a symptom score for each of the seven mental health problems. Children are assigned one of three categories for each health problem: likely absent, possible, and likely present (28). DSM-IV cut-offs were determined to identify which category the child fell into at the time of assessment (27). The metrological qualities of this assessment tool are good, since its reliability and validity are better than those of other traditional instruments for measuring children's mental health (28–31). Indeed, the study by Shojaei et al., conducted with a large sample of French children, showed a reasonable internal consistency of each DI diagnosis of the French version, with Cronbach's alpha coefficients ranging from 0.62 to 0.89 (31). This tool is currently available in 11 different languages (e.g., English, French, Spanish, Turkish) with voice-overs from native speakers and it combines different modalities (i.e., text, pictures, and voice-over) to display symptoms, decreasing the possible risk of translation errors and subsequent cultural differences in the interpretation of the item content. Moreover, the ethnicity of the character can be adapted to White, Hispanic, Asian, or African, and each character has a corresponding ethnic and gender-neutral name to optimize identification of the child (32).

In this present study, we only analyzed the total score of the DI. The total score was used as a continuous variable and varied between 0 and 94 (the maximum possible). High scores indicate poor mental health.

#### 2.2.2. Children's socio-demographic characteristics

The children's socio-demographic characteristics included the following: age (6–8 years/9–12 years) and sex (girl/boy) of the child, whether the child was born in France (yes/no), and whether the child is attending school (yes/no).

#### 2.2.3. Parent's socio-demographic and economic characteristics

The parent's socio-demographic and economic characteristics included the following: region of birth [Sub-Saharan Africa/Europe (including EU), Commonwealth of Independent States/another region of the world], the family structure (lived with a partner/ did not live with a partner), the education level (never been to school or primary school/secondary or higher education), the employment status (work/no work), the administrative status (French or regularized/not regularized), the health insurance status (general health insurance and complementary health insurance/general health insurance but no complementary health insurance/no general health insurance or complementary health insurance), the household food insecurity measured using the United States Household Food Security Survey (US HFSS) tool (33) (food security/light food insecurity/moderate food insecurity/severe food insecurity), and the difficulties with the French language (yes/no).

#### 2.2.4. Living conditions

The living conditions included the following: length of homeless time using the median as a threshold (homeless for 2.3 years or less/homeless for more than 2.3 years), number of moves since homeless using the median as a threshold (1.3 or fewer moves per year since homeless/over 1.3 moves per year since homeless), parent's social isolation (yes/no), whether the child has their own bed (the child sleeps in the same bed as his/her brothers and sisters/ the child sleeps in parents' bed/ the child sleeps on a mattress on the floor or other), and whether the child feels at home in his/her accommodation (yes/no).

#### 2.2.5. Children's health

The children's health variables included the following: child's hospitalized in the last 12 months (yes/no) and the child's body mass index (thinness/standard/overweight or obesity). The anthropometric measurements (weight and height of the child) were done by a nurse, in the presence of the mother, using scales and measuring sticks. The weight and height were then used to calculate the child's body mass index. Child's body mass index was calculated using the formula BMI = weight/height2 (kg/m2). The ENFAMS survey used the International Obesity Task Force (IOTF) curves to determine weight status (34, 35).

#### 2.2.6. Additional variables used

The additional variables included the following: the child's experience of bullying (yes/no), and maternal suicidal risk (yes/no).

### 2.3. Ethical approval

The ENFAMS survey was approved by the national authority for the protection of personal data collected on individuals (CNIL, n_Dr-2013-147), which is responsible for protecting citizens' data. It was also approved by two ethics committees (CPP, Ref 2012 02 06, 22/08/2012, and CCTIRS, n_12.471, 17/09/2012). Written informed consent to participate in the ENFAMS survey was obtained for all families interviewed.

### 2.4. Statistical analysis

Descriptive analyses were first performed to describe the characteristics of included children aged 6 to 12 years and their parents, and to estimate the prevalence of mental health disorders in each child, as assessed using the DI.

We developed a DAG (directed acyclic graph) to represent the relationships between the explanatory variables of interest, known confounding factors and the mental health of children, according to an etiological explanatory approach (Supplementary Figure S1). This DAG allowed us to select a subset of relevant variables to include in our final model. We then developed a multiple linear regression model on complete data with the DI total score as the dependent variable.

We have adjusted for the following factors: child's age and sex, parent's education level, length of homeless time, child's body mass index, and maternal suicidal risk. A mother's mental health can affect her child's through different pathways, both biological and social. Adverse experiences may influence the next generation through multiple pathways. For instance, epigenetics potentially explains the diverse pathways by which trauma and stress are transmitted to future generations (36). The relationship between a mother's mental health and her child's mental health can also be mediated by less engaged parenting and poor early childhood development. Early childhood development is critical for positive lifelong outcomes, including mental health, which further underscores the importance of addressing the mental health needs of mothers (37, 38). We adjusted the model for educational level because parents' education level is a well-known socio-economic factor that plays a significant role in childhood mental health (39). Independent of the house qualities, homeless families with low educational level may face to a lack of health literacy so they cannot provide appropriate support to or seek help for their children who struggle with mental problems, although they need it (40). We adjusted also the model for child's body mass index, because another recent study on ENFAMS data showed a high level of food insecurity in homeless families in the the Greater Paris area (41). Homeless children are more likely to be underweight than those who have homes, due to a lack of money or family resources to access adequate nutrition. Paradoxically, homeless children may also develop overweight and obesity because these are the main forms of malnutrition in homeless families. Finally, we adjusted on homeless duration time because previous studies (42) have shown that children who had been exposed to poverty for a long time had lower scores in developmental assessments that those exposed less time. Poverty, lack of health care access, parenting stress, and inadequate parenting practices are known to be associated with poor mental and developmental outcomes in homeless children, suggesting cumulative effects (43).

All statistical analyses (descriptive and explanatory) were performed on weighted data, using the “survey” package in R. The significance level was 0.05 (5%). R software Version 4.0.5. was used.

## 3. Results

### 3.1. Population and characteristics

**Figure 1:**
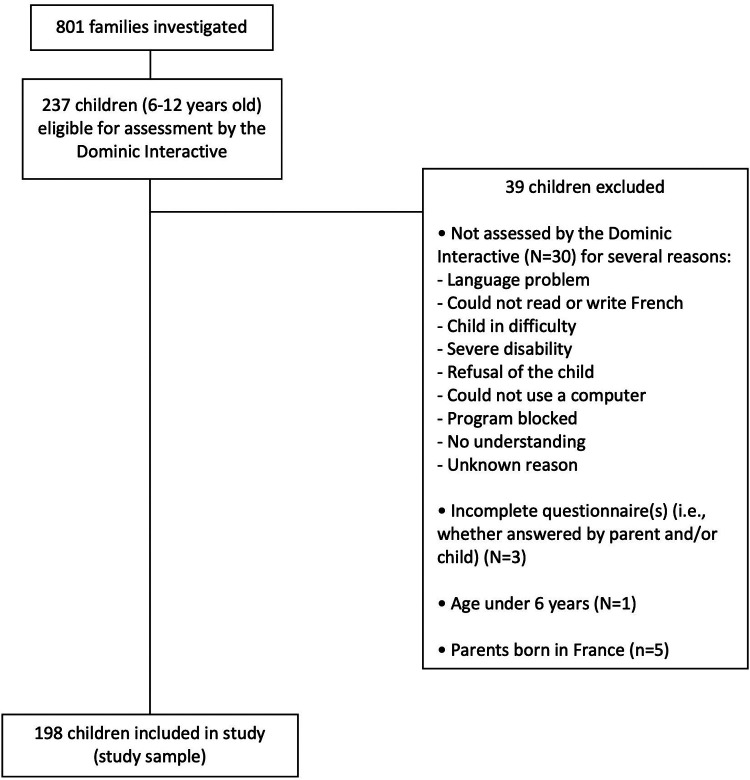
Flow chart of study sample, ENFAMS study, France, 2013.

Among the 237 children eligible for assessment by the DI in the ENFAMS study, 30 children were excluded because they could not be assessed by the tool for several reasons (Figure 1), 5 children were excluded because their parents born in France, 3 children were excluded because they are a lot of missing data in certain questionnaires, and one child was also excluded because their age was less than 6 years. In total, 39 children were excluded and therefore the descriptive analyses included 198 children. Excluded children were slightly younger, had less schooling than included children. They are also more likely to have parents who live with a partner, to have parents who do not have general health insurance and complementary health insurance, and to have parents who suffer from social isolation. These differences were statistically significant.

Children's and parent's characteristics are shown in Table 1.

**Table 1:** Children and parent's characteristics, ENFAMS study, France, 2013. (*N* = 198).

Variable	*N*	Weighted proportion %	95% CI
Children's socio-demographic characteristics			
*Age*	**198**		
6–8	109	56.5	[47.9; 65.0]
9–12	89	43.5	[35.0; 52.1]
*Sex*	**198**		
Girl	99	48.1	[39.9; 56.2]
Boy	99	51.9	[43.8; 60.1]
*Born in France*	**192**		
No	131	58.8	[49.2; 68.4]
Yes	61	41.2	[31.6; 50.8]
*Schooling*	**195**		
No	13	6.2	[2.8; 9.6]
Yes	182	93.8	[90.4; 97.2]
Parent's socio-demographic and economic characteristics			
*Region of birth*	**198**		
Sub-Saharan Africa	65	34.2	[25.1; 43.2]
Europe (including EU), Commonwealth of Independent States	77	35.6	[27.9; 43.3]
Another region of the world	56	30.3	[20.2; 40.3]
*Family structure*	**198**		
Lived with a partner	132	68.0	[59.6; 76.5]
Did not live with a partner	66	32.0	[23.5; 40.4]
*Education level*	**193**		
Never been to school or primary school	39	21.0	[13.8; 28.3]
Secondary or higher education	154	79.0	[71.7; 86.2]
*Employment status*	**197**		
Work	51	29.5	[21.5; 37.5]
No work	146	70.5	[62.5; 78.5]
*Administrative status*	**198**		
French or regularized	146	73.3	[66.1; 80.4]
Not regularized	52	26.7	[19.6; 33.9]
*Health insurance status*	**196**		
General health insurance and complementary health insurance	62	36.3	[27.9; 44.7]
General health insurance but no complementary health insurance	96	44.6	[36.0; 53.2]
No general health insurance or complementary health insurance	38	19.1	[12.3; 25.9]
*Household food insecurity*	**187**		
Food security	27	8.8	[5.2; 12.3]
Light food insecurity	64	38.3	[30.2; 46.5]
Moderate food insecurity	74	41.8	[32.5; 51.1]
Severe food insecurity	22	11.1	[6.0; 16.2]
*Difficulties with the French language*	**198**		
Yes	138	67.5	[57.6; 77.4]
No	60	32.5	[22.6; 42.4]
Living conditions			
*Length of homeless time*	**198**		
Homeless for 2.3 years or less	96	46.9	[38.6; 55.2]
Homeless for more than 2.3 years	102	53.1	[44.8; 61.4]
*Number of moves since homeless*	**191**		
1.3 or fewer moves per year since homeless	99	50.4	[41.0; 59.8]
Over 1.3 moves per year since homeless	92	49.6	[40.2; 59.0]
*Parent's social isolation*	**194**		
No	121	64.7	[56.5; 72.8]
Yes	73	35.3	[27.2; 43.5]
*The child has their own bed*	**196**		
Yes	92	38.8	[30.7; 47.0]
No, the child sleeps in the same bed as his/her brothers and sisters	43	25.2	[16.9; 33.5]
No, the child sleeps in parents’ bed	53	31.6	[23.6; 39.5]
No, the child sleeps on a mattress on the floor or other	8	4.4	[0.6; 8.1]
*The child feels at home in his/her accommodation*	**192**		
No	79	37.0	[28.9; 45.1]
Yes	113	63.0	[54.9; 71.1]
Children's health			
*Child's hospitalized in the last 12 months*	**192**		
No	173	90.0	[82.9; 97.0]
Yes	19	10.0	[3.0; 17.1]
*Child's body mass index*	**163**		
Thinness	13	5.7	[2.2; 9.2]
Standard	105	61.2	[48.8; 73.7]
Overweight or obesity	45	33.1	[20.5; 45.6]
Children's school experience			
*Child's experience of bullying*	**181**		
No	94	52.4	[44.1; 60.7]
Yes	87	47.6	[39.3; 55.9]
Maternal mental health			
*Maternal suicidal risk* ^a^	**198**		
No	174	88.8	[83.6; 94.0]
Yes	24	11.2	[6.0; 16.4]

^a^ Had thought about suicide, had attempted suicide, or had a suicide plan.

Values in bold indicate the total number of individuals for the variables studied. They represent the total number of observations for each variable measured.

### 3.2. Children's mental health

The DI scores (Table 2) indicated that 21.7% of our study sample possibly (“possible” category) had a mental health problem, and 3.6% probably (“likely present” category) had one. In terms of the latter dimension, the detailed breakdown was 12.8% for specific phobias, 13.8% separation anxiety disorder, 14.4% generalized anxiety disorder, 6.6% major depression/dysthymia, 3.3% oppositional defiant disorder, 11.2% conduct disorder, and 5.9% attention deficit hyperactivity disorder. The total mean score of the DI was 28.8 [SD = 17.5, 95% CI = (25.7; 31.9)].

**Table 2:** Average total score on the DI and average score for each mental health problem assessed, and distribution of the possibility and probability of mental health disorders in children aged 6 to 12 living in homeless families in the Paris area.

	*N*	Weighted proportion %	Weighted average (SD)	95% CI
Total (DI score) (94)			28.8 (17.5)	[25.7; 31.9]
Possible^a^ (42–63)	35	21.7		[14.9; 28.5]
Likely present^b^ (64–94)	13	3.6		[1.1; 6.1]
Internalizing				
Specific phobia symptoms (DI score) (9)			2.5 (2.0)	[2.2; 2.8]
Possible (3–4)	59	37.3		[27.3; 47.4]
Likely present (5–9)	35	12.8		[8.1; 17.4]
Separation anxiety symptoms (DI score) (8)			3.5 (1.8)	[3.2; 3.8]
Possible (5)	28	12.3		[6.3; 18.3]
Likely present (6–8)	33	13.8		[8.5; 19.1]
Generalized anxiety symptoms (DI score) (15)			6.6 (3.9)	[5.8; 7.4]
Possible (10–11)	21	8.7		[4.1; 13.3]
Likely present (12–15)	29	14.4		[7.1; 21.7]
Depression/dysthymia symptoms (DI score) (20)			6.3 (4.4)	[5.5; 7.1]
Possible (11–13)	22	10.9		[5.4; 16.4]
Likely present (14–20)	19	6.6		[2.6; 10.6]
Externalizing				
Opposition symptoms (DI score) (9)			2.5 (2.1)	[2.2; 2.8]
Possible (5–6)	23	15.6		[9.8; 21.4]
Likely present (7–9)	11	3.3		[0.7; 5.9]
Conduct problem symptoms (DI score) (14)			1.8 (2.8)	[1.3; 2.3]
Possible (3–5)	18	8.7		[4.3; 13.2]
Likely present (6–14)	23	11.2		[5.4; 16.9]
Hyperactivity–inattention symptoms (DI score) (19)			5.5 (4.4)	[4.7; 6.3]
Possible (11–13)	10	4.6		[1.9; 7.3]
Likely present (14–19)	17	5.9		[2.6; 9.2]

ENFAMS survey, France, 2013. (*N* = 198).

In our sample, the average total score of the Dominic Interactive was 28.8. 21.7% (*n* = 35) of children possibly had a mental health problem, while 3.6% (*n* = 13) probably had a mental health disorder.

SD, standard deviation; 95% CI, 95% Confidence Interval; DI, Dominic Interactive.

^a^ There is possibly a mental health problem.

^b^ There is probably (i.e., likely present) a problem.

### 3.3. Factors associated with children's mental health

Due to missingness data in some of the socio-demographic, economic, family, and health-related children and family factors, for assessing the potential association of such factors with the children's mental health outcome (total score for the DI), we used completed data analysis. Children excluded from the model due to missing data (*N* = 70) were not different from the children included in the model (*N* = 128), in terms of age, sex, socio-demographic and economic characteristics of parents (e.g., family structure, household food insecurity, education level, administrative status), living conditions (e.g., parental social isolation, the child has their own bed), children's and mother's health status (e.g., child hospitalized in the last 12 months, child's body mass index, maternal suicidal risk), and child's experience of bullying.

After adjusting for child's age and sex, parent's education level, length of homeless time, child's body mass index and maternal suicidal risk, the average total score for the DI of homeless children whose parents had an irregular administrative situation was 8.7 points higher than for those with French or regularized parents (95% CI: 1.3; 16.0, *p*-value = 0.022) (Table 3). Children who sleep in their parent's bed had an average total score for the DI 9.7 points higher than those who have their own bed (95% CI: 2.1; 17.4, *p*-value = 0.014). Children who had been hospitalized in the last 12 months had an average total score for the DI 11 points higher than those who were not hospitalized in the last 12 months (95% CI: 3.3; 18.7, *p*-value = 0.006). Finally, children who experienced bullying at school had an average total score for the DI 7 points higher than those who were not experienced bullying at school (95% CI: 1.6; 12.4, *p*-value = 0.012).

**Table 3:** Factors associated with mental health (as measured by the DI total score) among children aged 6 to 12 years living in homeless families in the Paris area, France.

	Weighted proportion %	Adjusted Coefficient^a^	95% CI	*p*
Parent's socio-demographic and economic characteristics				
*Family structure*				
Living with a partner (Ref)	69.0	-		
Not living with a partner	31.0	−1.3	[−8.3; 5.6]	0.702
*Administrative status*				
French or regularized (Ref)	76.5	-		
Not regularized	23.5	8.7	[1.3; 16.0]	0.022
*Household food insecurity*				
Food security (Ref)	8.3	-		
Low food insecurity	37.5	−5.7	[−17.7; 6.3]	0.343
Moderate food insecurity	46.0	−12.3	[−25.0; 0.4]	0.058
Severe food insecurity	8.2	−1.1	[−13.5; 11.4]	0.866
Living conditions				
*Number of moves since homeless*				
1.3 or fewer moves per year since homeless (Ref)	52.2	-		
Over 1.3 moves per year since homeless	47.8	−3.3	[−8.3; 1.6]	0.180
*Parent's social isolation*				
No (Ref)	67.4	-		
Yes	32.6	4.3	[−2.2; 10.8]	0.189
*The child has their own bed*				
Yes (Ref)	40.9	-		
No, the child sleeps in the same bed as his/her brothers and sisters	26.3	1.7	[−6.9; 10.2]	0.696
No, the child sleeps in parents’ bed	27.8	9.7	[2.1; 17.4]	0.014
No, the child sleeps on a mattress on the floor or other	4.9	4.9	[−9.1; 19.0]	0.482
*The child feels at home in his/her accommodation*				
No (Ref)	35.9	-		
Yes	64.1	1.2	[−4.9; 7.3]	0.689
Children's health				
*Child's hospitalized in the last 12 months*				
No (Ref)	87.9	-		
Yes	12.1	11.0	[3.3; 18.7]	0.006
Children's school experience				
*Child's experience of bullying*				
No (Ref)	50.7	-		
Yes	49.3	7.0	[1.6; 12.4]	0.012

ENFAMS survey, France, 2013. (*N* = 128).

95% CI: 95% Confidence Interval; p: *p*-value; Ref: Reference.

^a^ The results were adjusted for child's age and sex, parent's education level, length of homeless time, child's body mass index, and maternal suicidal risk.

## 4. Discussion

### 4.1. Main findings and interpretations

The prevalence of homeless children in the Greater Paris area (France) who possibly or probably had a mental health disorder in our sample was 25.3% and confirmed the previous findings (20) that highlighted the higher (SDQ) total scores higher in homeless children than those in the general population of France, (mean total score = 11.3 vs. 8.9). In comparison with a study conducted in France between 2000 and 2001 that used the DI to assess the mental health of children aged 6–11 years (27), we found a higher prevalence of the following disorders in our sample of homeless children: specific phobias (12.8% vs. 3.6%), separation anxiety disorder (13.8% vs. 5.5%), generalized anxiety disorder (14.4% vs. 2.8%), major depression/dysthymia (6.6% vs. 2.4%), conduct disorder (11.2% vs. 2.8%), and attention deficit hyperactivity disorder (5.9% vs. 5.5%). Our result were also consistent with the meta-analysis of Bassuk et al., where homeless school-aged children were significantly more likely to have a mental health problem than children with a home: overall, 24%–40% of homeless school-age had mental health problems requiring clinical evaluation, a rate 2 to 4 times higher than poor children aged 6–11 years in the National Survey of America's Families (44). The former grows up in a situation of great vulnerability, which is all the more detrimental to their development given their age. They experience and share the difficulties their parents face all the time. Families often encounter a lack of privacy, and schedules and rules necessary for shelter operations may conflict with family routines. Regular family mealtimes are challenging for families and sustained noise and crowding, common in some shelters, have been associated with less responsive parenting (45). The time and energy parents must dedicate to seeking employment or obtaining housing takes away from parenting and the maintenance of family routines and rituals (45). Our results showed that our French homeless population's characteristics also faced these issues (lack of privacy without own bed for the child, social isolation, food insecurity, etc.).

In our study, we identified several factors associated with a higher likelihood of mental disorders (as assessed using the DI score) in homeless children. Children whose parents had an irregular administrative situation on French territory (i.e., did not have official papers, had to undergo administrative procedures) had a total mean DI score 8.7 points higher than that of children whose parents were regularized. The issues of homelessness and housing deprivation in France have almost been linked to that of migration (46). This situation is related to the French public policy that brings the management of migratory flows and the world of social emergency closer together (46). In the recent period, the percentage of foreigners in the homeless population has risen, and probably due to several factors. Due to a lack of facilities and services designed specifically for migrants, there is a growing influx of migrants into emergency hostels and homeless shelters: temporary motel accommodation is seen as a solution for families with children, famously known as the “neither-nor”, as they were barely in a position to be regularized and scarcely in a position to be deported (46). Pervasive origin-based discrimination on the rental housing market is yet another contributing factor to migrant homelessness in France (47). Public policies on the admission of foreigners have become increasingly restrictive. In the majority of areas of daily life, social welfare, child welfare, health, housing, access to care are impacted by restrictive legal rules, that limit foreigners' full access to their fundamental rights. The health of the immigrant population, especially undocumented immigrants, is highly influenced by social determinants such as poverty, food insecurity, inadequate housing, lack of education, and difficulties in accessing health care (48). Our results stressed on this structural factor, understudied in previous studies: children of undocumented parents are at increased risk of poor physical and psychological health (48, 49). Waiting for the regularization of papers and having irregular status in the meantime is a difficult situation for the mental health of migrant parents, and leads to stress and anxiety about the future. The consequences, particularly for the mother, can have repercussions on the mental health and well-being of the child.

With regard to living conditions for mental health disorders, our study showed that sleeping in parent's bed was a risk factor. If co-sleeping could be a form of parenting in various cultures, this factor refers mainly to the lack of their own bed in welfare hotels and to the promiscuity in the housing. This result confirmed previous findings: permanent cohabitation with siblings and parents, overcrowding and the resulting lack of in individual or quiet space impacts children's physical and emotional development, schooling and intra-family relationships (50). Conflict in relationships may occur among siblings. Overcrowding can also impact physical health, with an increased risk of infection/transmission, which in turn can affect mental health (51, 52).

Indeed, our study shows precisely the negative influence on the child's mental health of a hospitalization in the last 12 months. Hospitalization for children means leaving their home and their caregivers, familiar figures and siblings and an interruption of their daily activities and routines. Due to their cognitive and emotional specificity and dependence on others, they are particularly vulnerable to stresses involved in adapting to their condition of illness and hospitalization (53). Moreover, anxiety-provoking experiences (such as hospitalizations) can affect children physical growth, motor, cognitive, or emotional and social development (54).

With regard to peer-based factors for mental health disorders, our study identified bullying at school as a factor. School is first and foremost a place of socialization, which is essential to a person's health and well-being. Some homeless children do not go to school. This lack of schooling is linked to four factors (55): frequent moves, which complicate the registration process; language difficulties and families' lack of knowledge of enrollment procedures; enrollment difficulties related to the absence of a place of residence; and the refusal of some municipalities to welcome children. For children not in school, socialization is limited. When children go to school, relationships with friends allow them to play and to confide their secrets. School is a daily and above all long-lasting meeting point for children (55). It provides a way to develop friendships, which are crucial for the development of long-term social behavior. The adults present in school are also an important support dimension, especially in terms of providing a sympathetic ear when students raise their concerns. School is a place where domestic violence can be detected and regular alerts can be given by professionals familiar with symptoms of suffering and abuse. A positive school experience is essential to the physical and well-being of the child (56). However, we did not test the effect of schooling on the mental health of children in homeless families in our model due to a lack of power. Indeed, the number of children not attending school was too small. Nevertheless, school can also be a place of bullying (55). Bullying by peers is a source of stress and not all children are resilient. School bullying is associated with many negative indicators, such as lower academic performance, lower satisfaction with school, and lower levels of attachment and engagement with school (57). These can have devastating consequences on a child's development and well-being in the short and long term, and may lead to school dropout. Among the homeless children who experienced school bullying in our sample, 10.3%, 6.9%, and 10.8% cited origin, living in a welfare hotel, and manner of speech are the reasons for being bullied. Children may feel ashamed of living in a welfare hotel and may feel stigmatized by their peers; the consequence is psychological suffering (14).

There is evidence based that children who are treated unfairly or discriminated against are more likely to have negative attitudes to school, lower levels of motivation and academic achievement, a higher risk of dropping out of formal education, experience of bullying and mental health problems (58). It is crucial for the adults present at school to tackle discrimination by promoting democracy, respect for human rights and citizenship, in order to promote well-being and educational success.

Our results were consistent with Roze's study, where children's difficulties were associated with parents' region of birth, residential mobility, children's health and overweight, the child's sleeping habits, the mother's suicide risk, the child's dislike of the family's accommodation and the child's experience of bullying (20).

### 4.2. Strengths and limitations

This study has several limitations. First, as the ENFAMS survey was cross-sectional, the direction of associations between some of the associated factors studied and the children's mental health outcome is difficult to determine. Second, although the DI is a valid and reliable tool, it does not provide a complete assessment of a child's mental health and do not assess the severity of mental symptoms. Children identify their internalized problems better than their externalized problems. The latter are better identified by parents or teachers. Third, the DI only examines the symptoms of mental health problems and not temporal aspects such as frequency, duration and age of onset. Fourth, DI is available in European French, French Canadian, European Spanish, Mexican Spanish, English and German but depending on the language of the respondent, the DI translation was used. However, for some oral languages (Lingala, etc.), it was not validated, which is a limitation of our study (28). Additionally, the DI is not specific to the very precarious or cultural contexts of homeless families. Finally, we probably under-estimate the mental health of homeless families as our sample did not included people living on the street, escapements, which could have a worsening impact on their mental health.

Our study also has strengths. First, it was based on data from the ENFAMS survey, which included a representative sample of homeless families in the Greater Paris area in 2013 with different cultural backgrounds. Homeless families are a hard-to-reach and under-researched population. ENFAMS made it possible to collect data on the mental health of homeless parents and children for the first time in the Greater Paris area. Second, ENFAMS used a robust sampling method to construct the sample. Taking into account the sample design and weighting in our analyses allowed us to describe all children in homeless families in the Greater Paris area, thus providing a representative overall picture of the mental health of these children, and not just the randomly drawn sample. Third, the survey was implemented by bilingual interviewers and a psychologist who jointly translated the questionnaires into 17 languages (9 of which were translated into written form). This ensured that the multicultural aspect that characterizes homeless families in the Greater Paris area would be taken into account. Fourth, the survey questionnaires were standardized and administered face to face, which made it possible to establish a relationship of trust between the interviewer and the respondent, thus limiting the risk of information bias. Fifth, the DI is a methodologically appropriate tool for use in epidemiological surveys of primary school-aged children in France (29). Direct assessment of children's mental health is a relevant methodological choice for research and epidemiological studies because it balances the child's own perception of his or her own symptoms with the perception of his/her health by other informants, such as parents. It allows the child to talk about internalized concerns more easily than traditional instruments (e.g., an interview by an adult clinician). In addition, the absence of interviewers and parents during the self-administered DI questionnaire significantly reduced social desirability bias, as the child was alone at the computer to answer the questions. Assessment of children's mental health is usually based on parents' information about their children's behavioral problems, so the prevalence of internalized problems may sometimes be underestimated. In our study, the child's own replies to the questionnaire helped to avoid this measurement bias. Furthermore, the interactive format of DI, where the questionnaire is similar to a video game, is appreciated by the children, and also helps to improve understanding of the questions.

One of the strengths of this study is that the child-based face-to-face questionnaire was adapted to children aged between 6 and 12 years old, enabling direct data collection from the children themselves, on various aspects, including social relationships, leisure activities, their relationship to their shelter, and experiences at school, including exposure to bullying. This is particularly important as children's exposure to bullying is rarely studied, especially in this population of vulnerable children, despite its potential impact on their mental health. These aspects are essential to gain a more thorough understanding of the children's experiences and how they may impact their mental health.

### 4.3. Conclusion

Our findings highlight the importance of the family environment with regard to homeless children's mental health and underline the need for prevention actions for these children, through better care in terms of accommodation, living conditions and health. Building on this point, it is also crucial to implanting more accessible culturally sensitive, family- and children-focused and trauma-informed mental healthcare and social support for families and children experiencing homeless (59–61). By implementing such measures, we could contribute to mitigating the negative mental outcomes associated with their housing insecurity and socioeconomic conditions. Furthermore, school bullying interferes with children's schooling and their mental health; awareness and intervention measures should be implemented to help reduce discriminatory behaviors and associated negative mental health impacts against children experiencing homelessness or housing instability in the school setting/environment (62, 63). More broadly, implementation of several recommendations already made by some of the authors of this article to the French “Defender of Rights” (64) to improve the daily lives of homeless families in hotels (65) would improve the mental health of children, such as guaranteeing a collective work and play room in each hotel, promoting access to recreational activities during school vacations or reducing hotel nomadism and the distance between the place of accommodation and school to promote continuity of schooling. Nevertheless, the priority remains to find other accommodation solutions than hotels, by promoting access to social housing, to centers dedicated to families or to emergency apartments.

The study sample comprised children 6–12 years old. However, more research needs to be done on homeless adolescents, another population that is vulnerable to mental health disorders and yet is under-researched.

## Data Availability

The datasets presented in this article are not publicly available due to limitations within the ethics approval but are available from the corresponding author on reasonable request. Requests to access the datasets should be directed to Stéphanie Vandentorren, Stephanie.VANDENTORREN@santepubliquefrance.fr.
