# Supplementary figures and images for: Housing and psychosocial factors associated with mental health in children aged 6–12 years from homeless families in the Greater Paris area, France: the ENFAMS cross-sectional study
Source: Front Child Adolesc Psychiatry. 2023 Jun 20;2:1136597. doi: 10.3389/frcha.2023.1136597 (PMC11732029; doi:10.3389/frcha.2023.1136597)

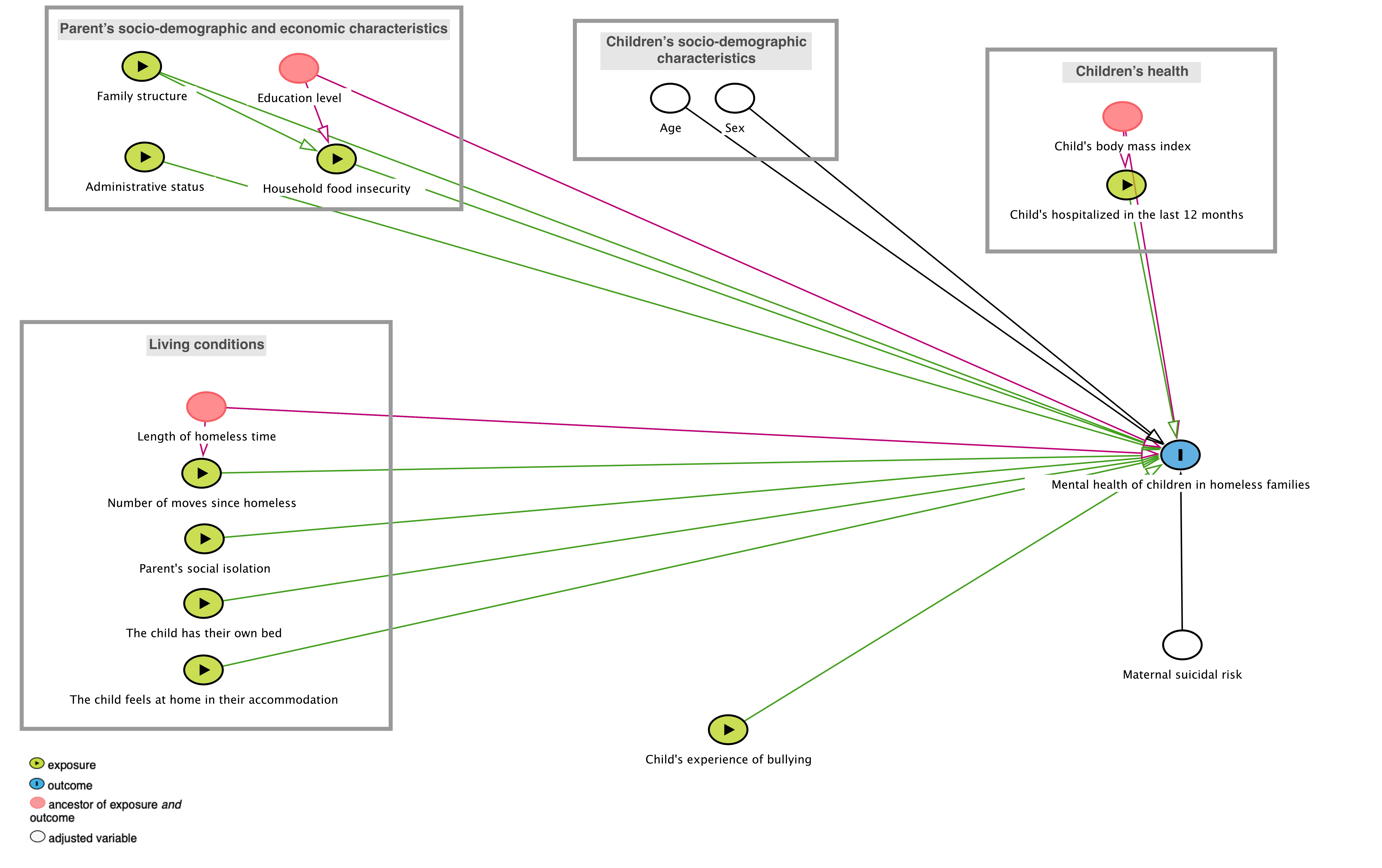

Supplement: Supplementary file 1 [file Image1.png]
